# C-Terminal Fluorescent Labeling Impairs Functionality of DNA Mismatch Repair Proteins

**DOI:** 10.1371/journal.pone.0031863

**Published:** 2012-02-14

**Authors:** Angela Brieger, Guido Plotz, Inga Hinrichsen, Sandra Passmann, Ronja Adam, Stefan Zeuzem

**Affiliations:** Department of Medicine I, University of Frankfurt/M., Frankfurt, Germany; University of Massachusetts Medical School, United States of America

## Abstract

The human DNA mismatch repair (MMR) process is crucial to maintain the integrity of the genome and requires many different proteins which interact perfectly and coordinated. Germline mutations in MMR genes are responsible for the development of the hereditary form of colorectal cancer called Lynch syndrome. Various mutations mainly in two MMR proteins, MLH1 and MSH2, have been identified so far, whereas 55% are detected within MLH1, the essential component of the heterodimer MutLα (MLH1 and PMS2). Most of those MLH1 variants are pathogenic but the relevance of missense mutations often remains unclear. Many different recombinant systems are applied to filter out disease-associated proteins whereby fluorescent tagged proteins are frequently used. However, dye labeling might have deleterious effects on MutLα's functionality. Therefore, we analyzed the consequences of N- and C-terminal fluorescent labeling on expression level, cellular localization and MMR activity of MutLα. Besides significant influence of GFP- or Red-fusion on protein expression we detected incorrect shuttling of single expressed C-terminal GFP-tagged PMS2 into the nucleus and found that C-terminal dye labeling impaired MMR function of MutLα. In contrast, N-terminal tagged MutLαs retained correct functionality and can be recommended both for the analysis of cellular localization and MMR efficiency.

## Introduction

DNA mismatch repair (MMR) is responsible for the correction of DNA replications errors and therefore essential for maintaining genomic stability and preventing tumor formation. Germline mutations in any of four MMR genes (*MLH1*, *MSH2*, *MSH6*, *PMS2*) cause the development of Lynch syndrome. MMR deficiency in tumors of patients with Lynch syndrome is characterized by microsatellite instability (MSI), which can be detected in more than 95% of associated carcinoma [1].

The initial step of human MMR is the recognition of mismatches by the heterodimer MutSα (MSH2 and MHS6) or MutSβ (MSH2 and MSH3). In the subsequent step the MutS complex recruits a MutL complex, most dominantly MutLα (MLH1 and PMS2) of which MLH1 is the essential component stabilizing its dimeric partner protein PMS2. Especially the last C-terminal alpha helix of MLH1 seems to be most important for PMS2 stabilization but also for the correction of mispairs as well as for checkpoint signaling in response e.g. to 6-thioguanine [2]. Beside this, MutLα is responsible for the recruitment of many downstream proteins essential for the repair process. Moreover, several different MMR dependent but also independent proteins have been published to interact with MutLα [3], [4], [5], [6], [7], suggesting its involvement in several other cellular processes like apoptosis and protein transport. Therefore, binding capacities as well as accessibility of MutLα's surface are supposed to be of utmost importance for the functional involvement of MutLα in these different processes.

Many *in vitro* data have been published using N-terminal [8], [9], [10], [11] or C-terminal [12], [13], [14], [15], [16], [17] fluorescent tagged MMR proteins. However, fluorescent labeling might have significant influence on the functionality of tagged proteins [18], [19], [20].

Therefore, we investigated the influence of N- or C-terminal dye labeling of MutLα on expression level, cellular localization and repair function. Using different combinations of coexpressed GFP- and Red-labeled or unlabeled MLH1 and PMS2 proteins, we compared expression level, cellular localization and the MMR functionality of these MutLα variants with the untagged MutLα.

## Results

### Single expression of MLH1 or PMS2 is significantly influenced by fluorescent labeling

In order to determine the influence of fluorescent labeling on single expressed MLH1 and PMS2 variants each of these proteins was transfected and expressed in HEK293T cells.

**Figure 1:**
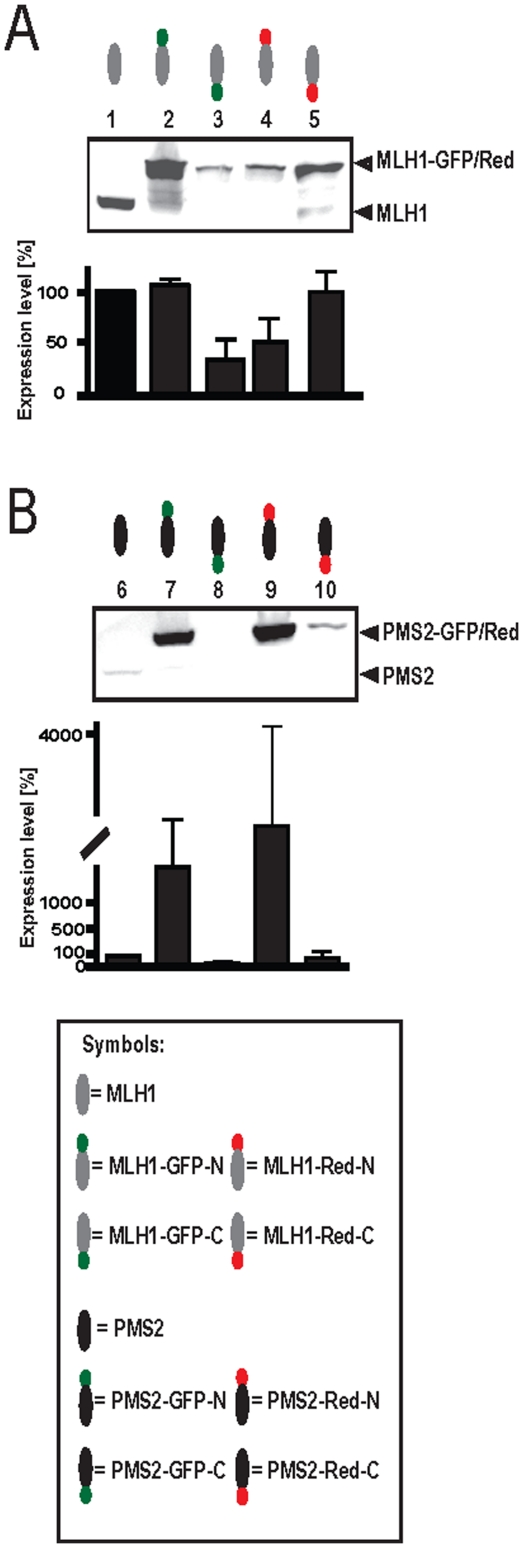
Dye tags influence single expression of MLH1 and PMS2. To determine the influence of fluorescent tags on single expressed MLH1 or PMS2 variants, HEK293T cells were transfected with different (A) MLH1 or (B) PMS2 constructs. Amounts of expressed proteins were assessed after Western blotting by measuring the signal intensities of protein bands with Multi Gauge V3.2 software. Graphs indicate the results (mean ±S.D.) of at least four independent experiments in which the proportion of protein expression using an unbiased method were presented. 1: MLH1 unlabeled; 2: MLH1-GFP-N; 3: MLH1-GFP-C; 4: MLH1-Red-N; 5: MLH1-Red-C; 6: PMS2 unlabeled; 7: PMS2-GFP-N; 8: PMS2-GFP-C; 9: PMS2-Red-N; 10: PMS2-Red-C.

As shown in Figure 1A, MLH1 is well expressed without coexpression of PMS2. However, N-terminal GFP (Figure 1A, lane 3) and C-terminal Red labeling (Figure 1A, lane 4) led to decreased expression levels.

In contrast, PMS2 (Figure 1B), normally unstable without coexpressed heterodimeric partner protein MLH1 [2], [22] and hardly expressed despite using overexpression-plasmid pcDNA3.1 (Figure 1B, lane 6), is well expressed and stable with N-terminal GFP or Red fluorescent labeling (Figure 1B, lane 7+9). However, C-terminal GFP or Red labeling resulted in very low or nearly undetectable expression of PMS2 (Figure 1B, lane 8+10), respectively.

### MutLα expression is influenced by fluorescent labeling

The influence of dye labeling on MutLα expression rate was analyzed by quantification of MLH1 and PMS2 levels 48 h after transiently cotransfection of different variants.

**Figure 2:**
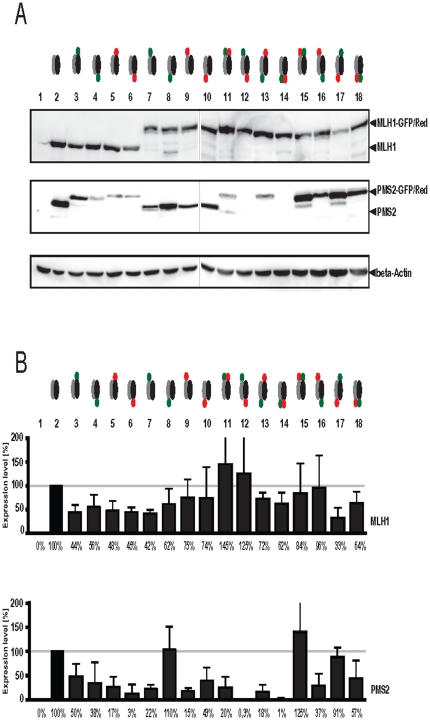
Influence of fluorescent labeling of MutLα on protein expression levels. To analyze the effect of fluorescent dyes on protein expression levels, HEK293T cells were transiently cotransfected with different MutLα constructs (see below) and (A) Western blot analysis was carried out after 48 h using anti-MLH1 or anti-PMS2, respectively, controlled by β-actin detection. (B) Amounts of expressed proteins were assessed by measuring the signal intensities of protein bands with Multi Gauge V3.2 software. Graphs indicate the results (mean ±S.D.) of at least four independent experiments in which the proportion of protein expression using an unbiased method were presented. 1: negative control (untransfected). 2: MLH1/PMS2 unlabeled; 3: MLH1/PMS2-GFP-N; 4: MLH1/PMS2-GFP-C; 5: MLH1/PMS2-Red-N; 6: MLH1/PMS2-Red-C; 7: MLH1-GFP-N/PMS2; 8: MLH1-GFP-C/PMS2; 9: MLH1-Red-N/PMS2; 10: MLH1-Red-C/PMS2; 11: MLH1-GFP-N/PMS2-Red-N; 12: MLH1-GFP-N/PMS2-Red-C; 13: MLH1-GFP-C/PMS2-Red-N; 14: MLH1-GFP-C/PMS2-Red-C; 15: MLH1-Red-N/PMS2-GFP-N; 16: MLH1-Red-N/PMS2-GFP-C; 17: MLH1-Red-C/PMS2-GFP-N; 18: MLH1-Red-C/PMS2-GFP-C. Symbols see Figure 1.

As shown in Figure 2, expression of fluorescent tagged-MutLα variants (Figure 2, lane 3–18) significantly differs from the untagged MutLα control (Figure 2, lane 2).

MLH1 expression in all single PMS2 labeled MutLαs (Figure 2, lane 3–6), in single MLH1 tagged (MLH1-GFP-N (Figure 2, lane 7) or MLH1-GFP-C (Figure 2, lane 8)) as well as in the double dye labeled MutLα variants, MLH1-GFP-C/PMS2-Red-C (Figure 2, lane 14) or MLH1-Red-C/PMS2-GFP-N (Figure 2, lane 17), was around 40–60% decreased compared to the untagged control. In contrast, expression of MLH1-GFP-N coexpressed with different Red labeled PMS2 (Figure 2, lane 11+12) was significantly higher in comparison to the control.

MLH1 expression of all other MutLα variants was about 70–80% compared to the control.

Looking at PMS2 expression, completely different results were detectable. Irrespective of whether MLH1 was tagged or not, C-terminal labeling of PMS2 with Red (PMS2-Red-C (Figure 2, lane 6, 12, 14)) led to dramatically decreased expression levels of around only 10% compared to untagged PMS2.

However, >100% of wild-type PMS2 expression was detectable when unlabeled PMS2 was coexpressed with MLH1-GFP-C (Figure 2, lane 8) or N-terminal labeled PMS2-GFP-N was coexpressed with MLH1-Red-N (Figure 2, lane 15).

In all other tested MutLα variants PMS2 expression was on average 30–60% compared to unlabeled PMS2.

### Subcellular localization of dye tagged MutLα

In order to analyze the influence of fluorescent proteins (GFP as well as Red) on subcellular protein localization, single expressed MLH1 or PMS2 as well as different coexpressed MutLα variants were analyzed in comparison to unlabeled MutLα using confocal laser microscopy.

**Figure 3:**
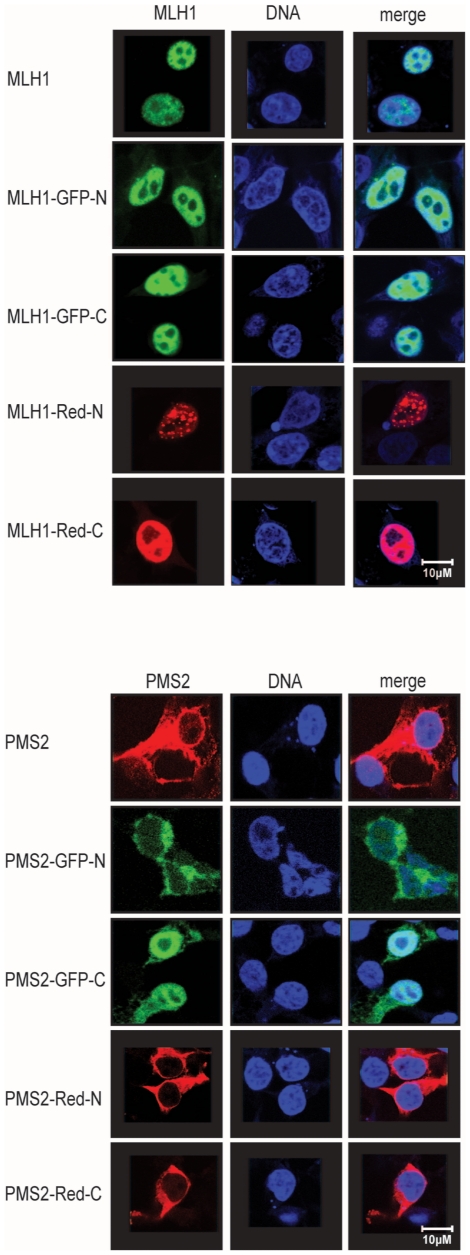
Subcellular localization of single expressed MLH1 and PMS2 variants. HEK293T cells were transfected with different MLH1 or PMS2 constructs as indicated to the left. MLH1 and PMS2 were visualized after 48 h using confocal laser microscopy. Nuclei were counterstained with TO-PRO-3 (middle column) after fixation and resulting overlay is shown in the right column. As a control, unlabeled MLH1 or PMS2 was transfected in parallel and visualized after 48 h using FITC-labeled goat anti-mouse IgG Alexa Fluor 488 for MLH1 detection (shown in green; left column) whereas unlabeled PMS2 was detected with the FITC-labeled goat anti-rabbit IgG Alexa Fluor 555. All used labeled MLH1 and PMS2 constructs showed the same cellular localization as the unlabeled form, with the exception of PMS2-GFP-C which was detected in the nucleus although all other constructs were located in the cytoplasm.

As shown in Figure 3, dye tagged single transfected MLH1 was, regardless of the orientation of the fluorescent tag, exclusively localized in the nucleus.

In contrast PMS2-GFP-N, PMS2-Red-C as well as PMS2-Red-N were, if transfected without heterodimeric partner protein MLH1, only detectable in the cytoplasm of transfected cells. However, single transfection of the PMS2-GFP-C variant led to strong nuclear localization of PMS2 in HEK293T cells.

All cotransfected MutLα variants were detected most dominantly in the nucleus (data not shown).

### MMR function is significantly impaired by fluorescent tags

MMR function of Lynch syndrome variants is commonly tested by *in vitro* MMR-assays [16], [28], [29]. Beside untagged, also dye-labeled proteins are used to analyze not only MMR functionality but also the influence of mutations on cellular localization and interaction in parallel [10], [17]. Hereby, several different MMR-fusion-proteins were utilized carrying e.g. either N- or C-terminal dye tags.

A dye-dependent change of protein function cannot be excluded especially for C-terminal protein labeling since the very sensitive MLH1/PMS2 interaction zone as well as the endonuclease function are located at the C-terminus and of great importance for the MMR function of MutLα [28]. Therefore, we determined the influence of the fluorescent tag orientation on the MMR functionality of MutLα.

**Figure 4:**
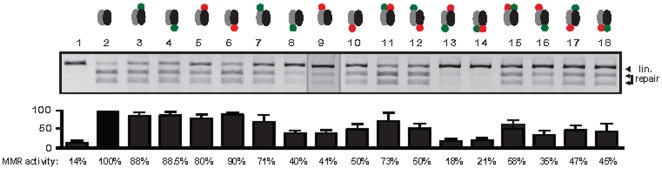
Influence of fluorescent tags on MMR activity of MutLα. HEK293T cells were transiently cotransfected with various labeled or unlabeld MutLα constructs and 48 h post transfection MMR activity of different MutLαs were assessed in vitro in parallel with unlabeled MutLα by quantifying the 3′-nick-directed correction of a G-T mismatch in a restriction site of a plasmid substrate as detailed in “Materials and Methods” and as previously described [10]. *In vitro* repair was scored on a 2-kb circular DNA substrate that contains an *EcoRV* site which is destroyed by a G-T mismatch. Upon repair of the G-T mismatch to an A-T base pair the intact *EcoRV* site together with an *AseI* site gives rise to a 0.8- and a 1.2-kb fragment, whereas unrepaired DNA is only linearized by *AseI* to a 2-kb fragment. Repair efficiency was assessed by measuring the signal intensities of linearized and digested vector with Bio-Rad Quantity One software using the “rolling ball” baseline correction. The signal intensity of the repair bands was divided by the intensity of all three bands. Repair efficiency of unlabeled MutLα was set at 100 percent and repair of fluorescent tagged MutLα was determined in relation to the wild-type sample that was expressed, processed and tested in parallel. Average repair values and standard deviations (±) were determined from four independent experiments. Single PMS2 tagged MutLαs, single MLH1-GFP-N tagged MutLαs as well as MLH1-GFP-N coexpressed with PMS2-Red-N or MLH1-Red-N coexpressed with PMS2-GFP-N were MMR proficient while all other tagged variants showed MMR deficiency. 1: mock control (untransfected). 2: MLH1/PMS2 unlabeled (positive control); 3: MLH1/PMS2-GFP-N; 4: MLH1/PMS2-GFP-C; 5: MLH1/PMS2-Red-N; 6: MLH1/PMS2-Red-C; 7: MLH1-GFP-N/PMS2; 8: MLH1-GFP-C/PMS2; 9: MLH1-Red-N/PMS2; 10: MLH1-Red-C/PMS2; 11: MLH1-GFP-N/PMS2-Red-N; 12: MLH1-GFP-N/PMS2-Red-C; 13: MLH1-GFP-C/PMS2-Red-N; 14: MLH1-GFP-C/PMS2-Red-C; 15: MLH1-Red-N/PMS2-GFP-N; 16: MLH1-Red-N/PMS2-GFP-C; 17: MLH1-Red-C/PMS2-GFP-N; 18: MLH1-Red-C/PMS2-GFP-C. Symbols see Figure 1.

All single PMS2 tagged (N- or C-terminal) MutLαs were still MMR proficient showing 80% or more MMR activity (Figure 4, lane 3–6).

In contrast, most single MLH1 labeled (with the exception of MLH1-GFP-N (Figure 4, lane 7) and most double fluorescent labeled MutLα-variants showed less than 50% MMR activity compared to wild-type MutLα. Hereby, MLH1-GFP-C coupled with PMS2-Red-C or PMS2-Red-N, respectively showed the most limited functionality. C-terminal GFP or N- and C-terminal Red MLH1 labeling with or without combination of fluorescent labeled PMS2 showed only ≤50% repair activity thus were MMR deficient.

In contrast, dye tagged MutLα, consisting of N-terminal tagged MLH1 and N-terminal fused PMS2 showed most MMR functionality whereby MLH1-GFP-N/PMS2-Red-N showed 70–85% MMR activity and therefore seems to be the most useful dye tagged MutLα variant.

## Discussion

Functional testing of MMR protein variants is of great importance to distinguish pathogenetic relevant mutations and non-pathogenetic polymorphisms detected in Lynch syndrome families [16], [30], [31]. Beside repair activity, also cellular localization and protein stability serve for the classification and dye tagged variants are frequently used to simplify MMR-protein analysis [2], [8], [16], [30]. However, a negative impact of fluorescent labeling on proteins' functionality might be present [18], [19], [20]. Therefore, we compared expression level, cellular localization and MMR activity of untagged and dye tagged MutLα variants and found significant changes in all parameters.

Expression levels of fluorescent labeled MutLαs significantly differ from unlabeled MutLα. As shown by our results, fusion of GFP and Red can dramatically affect protein stabilization. N-terminal fusion of dyes e.g. lead to stabilization even of single expressed PMS2 which is normally unstable without its heterodimeric partner. Our observation of this dye-dependent effect is in accordance to previously published data describing the large list of possible effects using fluorescent fusion proteins [32]. Thus, fluorescent tagged proteins do not seem to be useful for protein stability analysis.

However, differences in protein expression seemed to be without significant impact on MMR functionality of the heterodimer. Weak protein-expression, e.g. of tagged PMS2, did not automatically cause dramatic MMR decrease or vice versa, which is in accordance to Cejka *et al.* who detected that the amount of overexpressed MutLα is not the limiting factor in the MMR assay so that protein expression could be strongly reduced without functional impact [33].

Looking at the influence of dye labeling on MMR in detail, dye tags generally decrease repair function of MutLα even if only one partner protein was labeled. Hereby, labeling of MLH1 had a stronger restrictive effect than PMS2 tagging which illustrates the well known great importance of MLH1 for MutLα stability, transport and thereby MMR function [2], [22], [34].

**Figure 5:**
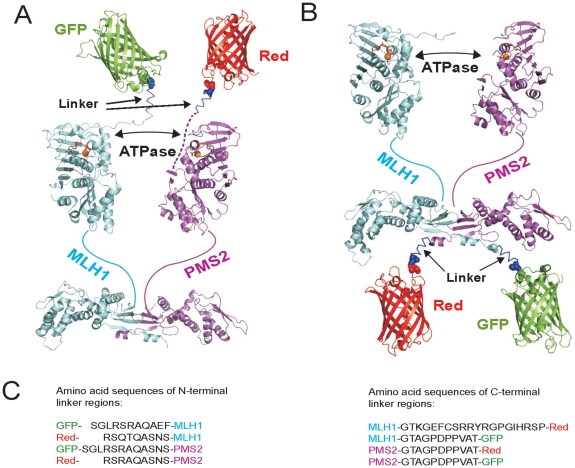
Putative three-dimensional structure models of fluorescent labeled MutLα. Using PyMol (Warren DeLano, http://www.pymol.org/), GFP or Red fluorescent proteins were attached to (A) N-termini of MLH1 and PMS2 or (B) C-termini of MutLα. C-terminal tags seem to hide the C-terminal region of MutLα and consequently might avoid DNA interaction. (C) Corresponding amino acid sequences of linker regions are shown. The dashed line between PMS2 and the N-terminal fluorescent tag illustrates a putative α-helix (unknown structure) of the first thirty amino acids of PMS2.

Fluorescent tags fused to the C-terminus of MutLα components, most obvious GFP-fusion to the C-terminus of MLH1 entirely impair MMR function of the heterodimer. However, exclusively N-terminal tagged MutLαs showed MMR proficiency which emphasizes that the N-termini of MLH1 and PMS2 are much more tolerant to dye protein extension than their C-termini. Since MutLα was described to coordinate protein-protein interaction during the MMR process via its N-terminus [35] the observed tolerance of this domain to fluorescent dyes are explainable. In contrast, we assume that C-terminal tags might lead to incorrect interaction of the heterodimeric partners or to a strong hindrance of the MutLα radius of action which would fit well to the great importance of the last C-terminal alpha helix of MLH1 for nucleotide binding and correction of mispairs [2]. Moreover, this postulation is enforced by our computer modeling of a three-dimensional structure of dye labeled MutLα (Figure 5) consisting of previously published homology models of MLH1 and PMS2 [24], [25], [26], [27] and structures of GFP or Red (PDB number: 2WSN (GFP); PDB number: 1G7K (Red)). C-terminal interaction of MLH1 and PMS2 fix the C-terminus of the heterodimeric complex and additional adhesion of dye complexes might hide the functional surface and lead to the observed impaired ability for MMR.

Although GFP and Red show only marginal differences in structure, GFP overall had less impact on MutLα function than Red.

Looking at the influence of fluorescent fusion on cellular protein localization we found one dye-dependent incorrect protein transport. While PMS2 is normally not able to enter the nucleus without dimeric partner protein MLH1 [2], [11], [22], [34] C-terminal labeling with GFP (although PMS2-GFP-C was very weak expressed) wrongly enables this protein to be shuttled into the nucleus. This nuclear localization of single expressed C-terminal dye tagged PMS2 was previously detected by Raevaara *et al.* as well as Leong *et al.* who used a PMS2-GFP-C or a PMS2-Red-C construct, respectively [13], [15]. Leong and coworkers thereupon postulated a weak nuclear import ability of PMS2. However, we presume that nuclear import of C-terminal GFP tagged single expressed PMS2 is only a dye-depending phenomenon and might lead to an incorrect interpretation of mutational influence of MLH1 on PMS2 transport mechanism.

In order to overcome the described problems, one might consider switching the tags to any other of the numerous fluorescent dyes available. However, due to high structural similarities between all these dyes we do not expect relevant functional differences compared to those generated with GFP or Red. Impaired repair function of MutLα by C-terminal labeling has to be assumed using any of these fluorescent dyes.

In summary, only fusion of fluorescent proteins to the N-termini of MLH1 and PMS2 enable correct functionality and cellular localization of MutLα and are recommended for testing of MutLα variants without restrictions.

## Materials and Methods

### Cells and Cell Transfection

HEK293T cells, obtained from Dr. Kurt Ballmer (Paul Scherer Institute, Villingen, Switzerland) were grown in Dulbecco's Modified Eagle Medium (DMEM) with 10% FCS. HEK293T is a clone of HEK293 that expresses the SV40 large T antigen. As previously published, MutLα is not expressed in HEK293T [21].

Transfection and cotransfection of HEK293T cells were carried out as described previously [7]. In brief, HEK293T were transfected at 50–70% confluence with expression plasmids (1 µg/ml, respectively) using 10 µl/ml of the cationic polymer polyethylenimine (Polysciences, Warrington, PA; stock solution 1 mg/ml). 48 h post-transfection whole cell extract was prepared for Western blot analysis, MMR assay or confocal laser microscopy.

### Plasmids

Used pcDNA3.1+/MLH1, pcDNA3.1+/PMS2, pECFP-C1/MLH1 (MLH1-GFP-N) and pDSRed-C1/PMS2 (PMS2-Red-N) expression plasmids were described previously [10], [22]. pECFP-N1- or pDSRed-N1-MLH1 (MLH1-GFP-C or MLH1-Red-C) and pECFP-N1- or pDSRed-N1-PMS2 (PMS2-GFP-C or PMS2-Red-C) were generated by subcloning of wild-type MLH1 [22] or PMS2 [22] using *EcoRI* and *KpnI* (DNA-cloning, Hamburg, Germany) into pECFP-N1 or pDSRed-N1 (Clontech Lab., Palo Alto, CA, USA). All used dye labeled MMR proteins are illustrated in Figure 1, confirmed by sequencing and reading frames were corrected using site-directed mutagenesis, if necessary.

Oligonucleotides were purchased from Sigma-Aldrich (Munich, Germany).

### Antibodies, Western blot analysis, and protein quantification

Anti-MLH1 (G168-728) was obtained from Pharmingen (BD Biosciences, United States), anti-PMS2 (E-19) was from Santa Cruz Biotechnology (Santa Cruz, CA), and anti-β-Actin (Clone AC-15) was purchased from Sigma (Sigma-Aldrich, Munich, Germany). FITC-labeled goat anti-mouse IgG Alexa Fluor 488 as well as FITC-labeled goat anti-rabbit IgG Alexa Fluor 555 were obtained from Invitrogen (Darmstadt, Germany).

Proteins were separated on 10% polyacrylamide gels, followed by Western blotting on nitrocellulose membranes and antibody detection using standard procedures.

The band intensity of proteins was quantified using Multi Gauge V3.2 program (Fujifilm, Tokyo, Japan).

All experiments were performed in quadruplicate.

### Confocal Laser Microscopy

To analyze the influence of fluorescent labeling on cellular localization of MLH1 or PMS2 proteins, HEK293T cells were transiently transfected with unlabeled, GFP- or Red-labeled variants. Briefly, HEK293T cells were spread on coverslipes to 50% confluence 4 h prior transfection and transfected with expression plasmids (1 µg/ml, respectively) using 10 µl/ml of the cationic polymer polyethylenimine (Polysciences, Warrington, PA; stock solution 1 mg/ml). After 48 h, cells were prepared for confocal laser scanning microscopy essentially as described previously [10]. In brief, all cells transfected with fluorescent labeled as well as those transfected with unlabeled proteins were washed twice with PBS, fixed for 10 min in 3% formaldehyde in PBS, followed by permeabilization with 0.1% Triton X-100 for 5 min. Furthermore, cells transfected with unlabeled MLH1 or PMS2 were incubated for 1 h with the primary antibodies against MLH1 (1∶1000) or PMS2 (1∶1000) at room temperature, respectively. MLH1 antibody binding was detected with a FITC-labeled goat anti-rabbit IgG Alexa Fluor 488 (1∶1000) whereas PMS2 antibody binding was detected with FITC labeled goat anti-rabbit IgG Alexa Fluor 555. Before mounting TO-PRO-3 (Invitrogen, Germany) diluted 1∶1000 in PBS was added to all coverslipes for 1 h at room temperature to counterstain nucleic acids and coverslipes were washed again with PBS.

After that, samples were mounted with ProLong Gold (Invitrogen, Germany) and examined using a Leica TCS-NT confocal microscope (Leica Lasertechnik GmbH, Heidelberg, Germany). Control for subcellular localization was carried out using unlabeled MutLα and mock control for fluorescent labeled MutLα was determined with the pECFP-C1/-N1- and pDSRed-C1/-N1- vectors.

### MMR Assay

To analyze the consequences of GFP or Red labeling different MutLα variants were tested *in vitro* for MMR ability as described before [23]. Briefly, 50 µg of HEK293T nuclear extract, which is deficient in mismatch repair [21] was supplemented with 5 µg whole protein extract from HEK293T cells expressing recombinant MutLα constructs. A substrate plasmid bearing a G-T mismatch within an *EcoRV* restriction site was added to these protein compositions, which is restored when repair occurs directed by a 3′ single-strand nick at a distance of 83 bp to the mismatch. Reactions were incubated at 37°C for 20 min and terminated with 50 µl stop-buffer (24 mM EDTA pH 8.0, 0.7% SDS, 2 µg/µl proteinase K) by an additional incubation for 15 min at 37°C. Plasmids were extracted from the reaction mixture by phenol-chloroform extraction and purified by ethanol co-precipitation with tRNA. Subsequent digestion with *EcoRV* as well as *AseI* produced two smaller fragments besides the linearized vector (which is generated by *AseI* digestion) when repair was successful. Restriction digests were separated on 2% agarose gels, stained with ethidium bromide and bands were quantified using Quantity One Software v4.6.1 (Bio-Rad, Hercules, CA, USA). Repair efficiency of unlabeled MutLα was set at 100 percent and repair of fluorescent tagged MutLα was determined in relation to the wild-type sample that was expressed, processed and tested in parallel.

Although the amount of mismatched plasmid DNA present in parallel incubations is always identical (aliquoted from one master mix), phenol-extraction/ethanol precipitation of the processed plasmid can show differences in recovery, and therefore the overall DNA amounts can differ from lane to lane. However, repair efficiency is measured as quotient of the intensities of bands indicating repair and the sum of all band intensities and therefore gives an accurate repair value independent of the amount of DNA actually recovered during plasmid extraction.

All experiments were performed in quadruplicate.

### Structural modeling of fluorescent labeled MutLα

Structural modeling of MutLα as well as three-dimensional structures of GFP- or Red-proteins has been described previously [24], [25], [26], [27]. Protein structures were visualized using PyMol (Warren DeLano, http://www.pymol.org/).
